# An integrative review and evidence-based conceptual model of the essential components of pre-service education

**DOI:** 10.1186/1478-4491-11-42

**Published:** 2013-08-28

**Authors:** Peter Johnson, Linda Fogarty, Judith Fullerton, Julia Bluestone, Mary Drake

**Affiliations:** 1 Jhpiego, 1615 Thames Street, Baltimore, MD 21231-3492, USA; 2 Independent consultant- 7717 Canyon Point Lane, San Diego, CA 92126-2049, USA

**Keywords:** Pre-service education, Conceptual model, Outcomes, Impact

## Abstract

**Background:**

With decreasing global resources, a pervasive critical shortage of skilled health workers, and a growing disease burden in many countries, the need to maximize the effectiveness and efficiency of pre-service education in low-and middle-income countries has never been greater.

**Methods:**

We performed an integrative review of the literature to analyse factors contributing to quality pre-service education and created a conceptual model that shows the links between essential elements of quality pre-service education and desired outcomes.

**Results:**

The literature contains a rich discussion of factors that contribute to quality pre-service education, including the following: (1) targeted recruitment of qualified students from rural and low-resource settings appears to be a particularly effective strategy for retaining students in vulnerable communities after graduation; (2) evidence supports a competency-based curriculum, but there is no clear evidence supporting specific curricular models such as problem-based learning; (3) the health workforce must be well prepared to address national health priorities; (4) the role of the preceptor and preceptors’ skills in clinical teaching, identifying student learning needs, assessing student learning, and prioritizing and time management are particularly important; (5) modern, Internet-enabled medical libraries, skills and simulation laboratories, and computer laboratories to support computer-aided instruction are elements of infrastructure meriting strong consideration; and (6) all students must receive sufficient clinical practice opportunities in high-quality clinical learning environments in order to graduate with the competencies required for effective practice. Few studies make a link between PSE and impact on the health system. Nevertheless, it is logical that the production of a trained and competent staff through high-quality pre-service education and continuing professional development activities is the foundation required to achieve the desired health outcomes. Professional regulation, deployment practices, workplace environment upon graduation and other service delivery contextual factors were analysed as influencing factors that affect educational outcomes and health impact.

**Conclusions:**

Our model for pre-service education reflects the investments that must be made by countries into programmes capable of leading to graduates who are competent for the health occupations and professions at the time of their entry into the workforce.

## Background

Contemporary pre-service education (PSE) for the health occupations and professions focuses on the acquisition of competencies that are needed immediately upon entry into the workforce - the knowledge, skills, attitudes and behaviours necessary for safe and effective practice [1]. With decreasing global resources, a pervasive critical shortage of skilled health workers in low- and middle-income countries, and growing disease burden in many countries, the need to maximize the effectiveness and efficiency of education and training for health workers has never been greater. International public health agencies, donors and governments are increasingly interested in making long-term investments to improve pre-service education.

Pre-service education (PSE) is used in this article to refer to the curriculum of studies that prepares a health provider for entry into practice of a health profession. (The term could also encompass preparation for practice of selected health occupations (pre-technical or pre-vocational education); however, we do not focus on these health provider cadres in this particular article.) High-quality PSE is both influenced by and facilitated by the interaction of several factors that promote competence within a defined scope of practice and within a collaborative, client-oriented health system that reflects local country needs. Jhpiego, an international non-governmental organization with many programmes supporting education for health workers, conducted an integrative review of the literature [2] to examine what is presently known about the essential components of pre-service education. An integrative review differs from a systematic review in that it allows inclusion of diverse methodologies (including both experimental and non-experimental studies), and takes a broader range of studies (both theoretical and empirical) into consideration [2]. We used this literature synthesis to develop a conceptual model that describes essential components of PSE and links them to desired outcomes and public health systems impact. The model will enable technical advisors, policymaking bodies and donors at the global and country levels to maximize the returns on their efforts to strengthen PSE systems. Specifically, the model can be used as a framework for prioritizing investments and as a guide for programme monitoring, evaluation and research to create a comprehensive evidence base for PSE strengthening.

## Methods

### Literature review methodology

Three sources were used for this review: electronic peer-reviewed literature, a ‘hand search’ of PSE bibliographies, and a focused literature search. First, the electronic peer-reviewed literature from 2000 to 2011 was searched within multiple health science databases (Pubmed, Medline, Cinahl, Google Scholar). Primary search terms focused on primary health providers in the global community (for example, physicians, nurses, midwives, clinical officers). Secondary search terms broadened the search to include a variety of specialties in which the health workers might be additionally prepared. Secondary search terms also included education, evaluation and regulation terms as well as aspects of the intended outcomes of pre-service education.

Two reviewers, working independently, scanned the titles and abstracts of the resulting 1,742 articles for their potential value to the issue of pre-service education. Articles were excluded from consideration if they: (1) reported on issues that were unlikely to have wide, generalizable application (for example, reports on a one-time or short-term experience at a single educational site); (2) were very narrow in focus (for example, an assessment of changes in courses or programme policies at a single institution, without intent to create models for replication); or (3) reported very limited sample sizes. In addition, articles that reported on postgraduate training events (continuing professional education and in-service education) were excluded, so that the PSE review would focus as precisely as possible on the essential elements and methods of pre-service education. Opinion pieces, editorials, commentaries, book chapters and project reports (grey literature) were also excluded. This yielded 124 articles with an explicit focus on pre-service education.

Second, a hand-search of existing PSE bibliographies yielded an additional 14 studies. Third, a focused literature search was conducted to address gaps in the previous search in specific topic areas that were underdeveloped. This search yielded an additional 82 unduplicated studies.

**Figure 1:**
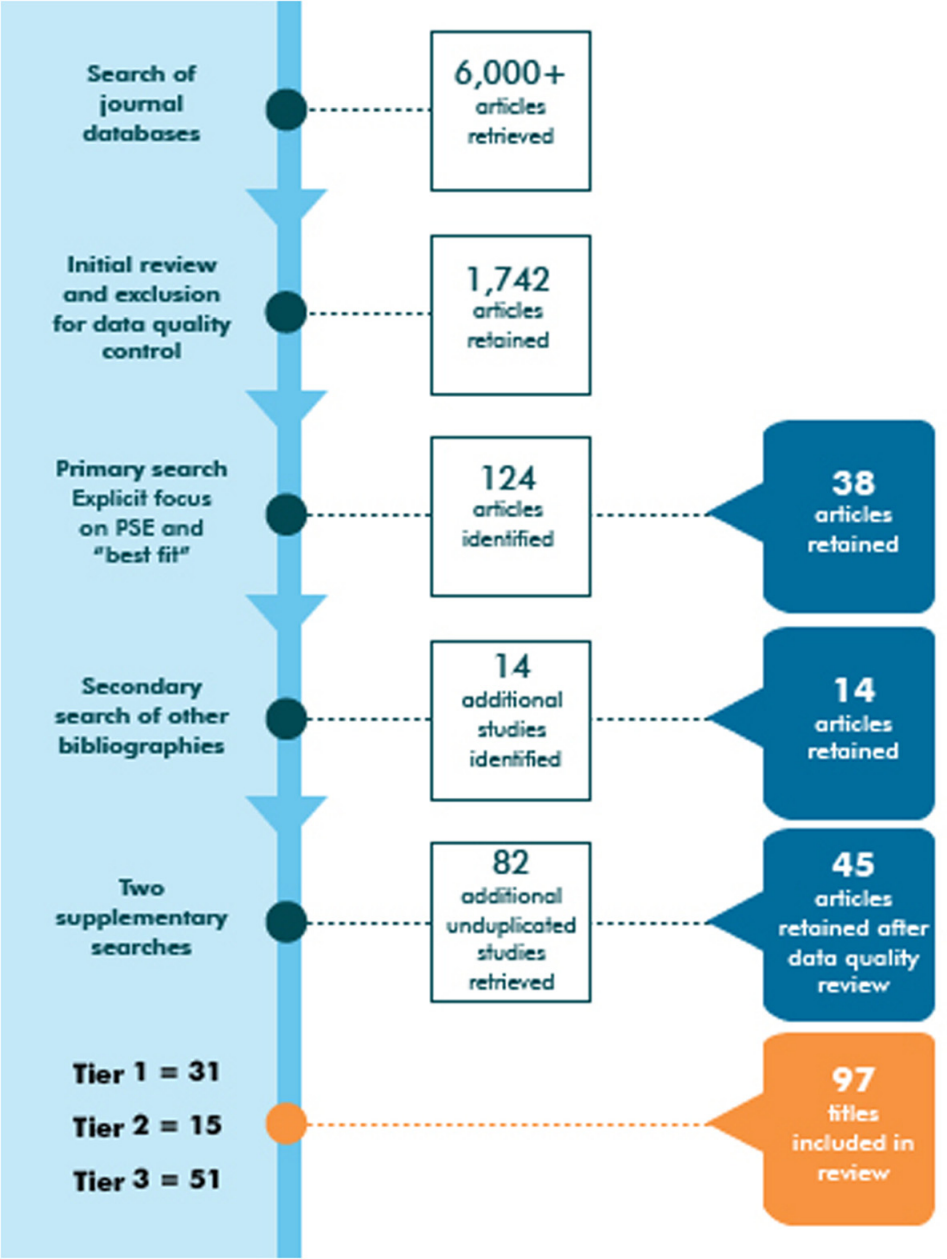
Article inclusion process.

Articles were graded using a commonly accepted categorization scheme (the SORT approach), which is recommended for grading the quality of evidence published in the medical literature [3]. Table 1 depicts these grading criteria and the three quality tiers (the SORT tiers) that are represented by these evidence grades. A total of 97 articles (38 from the first source, 14 from the second source, and 45 from the third source) were retained after this process. Of these, 31 were graded as Tier 1, 15 as Tier 2, and 51 as Tier 3. Each of the 97 articles was also graded with respect to its focus on pre-service education, using the approach adopted for the *Best Evidence in Medical Education* reviews [4,5]. Figure 1 depicts the inclusion and exclusion process.

**Table 1:** Grading criteria

**Design**	**Types of groups**	**Literature grade**	**Tier**
Meta-analysis		1	1
Systematic or integrative review of literature		1	
Experimental	Between subjects (experimental and control)	2	
	Within subjects (cross-over)	2	
Quasi-experimental	Non-equivalent control group	3	2
	Repeated measures	3	
Pre-experimental	Comparison group	4	
	Pre-test/post-test	4	
	Post-test only	5	
Descriptive studies	Retrospective	6	3
	Case series, correlation	6	
	Prospective/cross-sectional	6	
	Literature review (descriptive)	6	
Qualitative studies		7	

### Conceptual model

**Figure 2:**
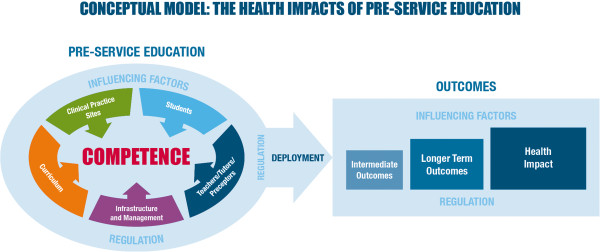
Pre-service conceptual model.

Five essential inputs to a comprehensive PSE system and several key influencing factors emerged as a result of the literature review. We created a conceptual model that depicts these five broad categories of inputs: students, curriculum, teachers/preceptors, infrastructure and management, and clinical practice sites (Figure 2). The expected educational output of these five elements is a competent graduate.

When competent graduates are deployed, we expect that their work will contribute to certain outcomes. We expect that they will implement life-saving practices and demonstrate professional behaviours, perform those practices to standard, and make a public health impact by contributing to decreases in morbidity and mortality. Several elements of the political, policy, social and workplace environments may either augment or impede the preparation of qualified graduates who are able to provide high-quality health services. Professional regulation emerges as an influencing factor of special significance.

## Findings

Table 2 offers illustrative excerpts of findings from this integrative review. The findings are summarized below according to the model.

**Table 2:** Illustrative outcomes from the pre-service education experimental and descriptive literature

**Author(s) and Years**	**Country**	**Cadre**	**Subjects (research) ( *n *)**			**Literature grade **^**a**^	**Kirkpatrick level **^**b**^	**Summary**
			**Studies (reviews) ( *n *)**					
			**Total**	**Intervention**	**Control or comparison**			
*Qualified student body*								
Grumbach & Chen, 2006 [6]	USA	Medical students	661	265	396	4	2B	Applicants from minority and disadvantaged groups who participated in post-baccalaureate pre-medical programmes were significantly (*P* = 0.001) more like to gain admission. Participants demonstrated higher MCAT scores (odds ratio 8.06, CI, 4.08 – 9.71)
Rolfe et al., 2004 [7]	Australia	Medical students	498	344	154	4	1B	Graduates from prior 16 years were surveyed. Students admitted directly from high school were equally successful to those admitted following university education. No significant differences were demonstrated in terms of academic performance (*P* = 0.83) or postgraduate practice sites in urban or rural settings (*P* = 0.46)
*Student support and retention*								
Santee & Garavalia, 2006 [8]	N/A	Nursing students	20			1	2B	This systematic review concluded that peer tutoring had positive impact on academic performance, compared to no tutoring. Peer tutoring performed slightly better than faculty tutoring. However, few higher quality studies could be identified for inclusion in the review
Pariyo et al., 2009 [9]	N/A	Students of various health professions	2			1	1B	This Cochrane review concluded that a student support package that included social and academic support and mentorship, and career guidance, resulted in an increase in the number of minority students who enrolled and graduated from health training institutions
*Competency-based curriculum*								
Tamblyn et al., 2005 [35]	Canada	Medical students	751	151	600	2	4B	Three comparison cohorts of medical graduates who had studied within a traditional curriculum were compared to one cohort of graduates of a problem-based-learning (PBL) focused curriculum. Assessment of their performance in practice on measures of preventive care, continuity of care, diagnosis and management were significantly higher for the PBL cohort (for example, a four-fold increase in disease specific prescribing compared to prescribing for symptom relief)
*Curriculum aligned with national health priorities*								
Kaye et al., 2010 [11]	Uganda	Medical students	60	30	30	3	1B	Students who completed a PBL curriculum, including experiential training in rural health settings, were compared to a cohort who graduated from a traditional curriculum. The PBL experience significantly influenced the choice to work in a rural/underserved area
Laven & Wilkinson, 2003 [12]	N/A	Medical students	12			1	1B	This systematic review addressed the effect of rural background on willingness to enter rural practice identified a strong association. Odds ratios exceeded 2.0. Rural undergraduate training had a similar positive influence
Longombe, 2009 [13]	Democratic Republic of the Congo	Medical school graduates	163	43	120	4	1B	Graduates of a medical school located in a rural area were compared to graduates of an urban-located school. Almost all (97.7%) of the graduates from the rural-located school were employed in the province in which they were educated; the majority (81.4%) in rural areas. Urban graduates were dispersed throughout the country, but only 23.7% were practicing in rural areas
*Teachers/tutors competent in technical areas*								
Steinert el al., 2006 [14]	N/A	Faculty of various health professions	53			1	1A	This systematic review concluded that faculty development activities (seminars, workshops, short-courses) are highly valued and lead to learning and behaviour change, as well as better teaching performance
							1B	
							2A	
							2B	
							3	
							4B	
*Competent preceptors*								
Udlis 2008 [15]	N/A	Nursing students	16			1	2A	This integrative review indicated that participation in a one-to-one preceptorship programme increased role conception and performance. However, no evidence was found that the preceptorship experience promoted critical thinking, clinical competence or improvement in licensing examination pass rates
							2B	
*Enhanced learning activities*								
Cook et al., 2008 [16]	N/A	Students of various health professions	201			1	1B	This systematic review compared Internet-based education to no intervention on outcomes addressing knowledge, skills, learner behaviours and patient effects. Data synthesis shows that Internet-based learning - when compared with no intervention - is associated with large positive effects (*P* <0.001 for each) but the effectiveness of Internet-based learning appears to be similar to that of traditional methods
							2B	
							3	
Cook et al., 2011 [17]	N/A	Students of various health professions	609			1	1B	A total of 137 randomized studies, 67 two-group comparisons, and 405 pre-test/post-test studies concluded that in comparison with no intervention, technology-enhanced simulation training in health professions education is consistently associated with large effects for outcomes of knowledge, skills and behaviours (all effect sizes >1), and moderate effects for patient-related outcomes (effect size 0.50)
							2B	
							3	
							4B	
*Multidisciplinary learning experiences*								
Reeves et al., 2010 [18]	N/A	Students of various health professions	6			1	1A	A Cochrane review identified six studies (randomized controlled trials (RCT) or experimental studies on inter-professional education (IPE)); however, because component activities and measured outcomes were not consistent among the studies, comparison was limited. All six studies were favourable for the effect of IPE on student learning and clinical behaviours
							1B	
							1C	
							1D	
*Varied clinical practice sites*								
Dornan et al., 2006 [19]	N/A	Students of various health professions	73			1	1A	This systematic review concluded that having practical experience in clinical sites early in the curriculum of studies was motivating for students, helped them be more confident with patients; less stressed, more self-reflective, and enhanced their professional identity
							1B	
							2A	
							2B	
							3	
Littlewood et al., 2005 [20]	N/A	Medical students	73			1	2A	This systematic review indicated that early immersion into clinical practice fostered more positive attitudes toward studies, greater understanding of subject matter, enhancement of clinical skills, and had benefits on organizations, populations and patients
							3	
							4B	

N/A, not applicable.

^a^Literature grades: 1, metaanalysis, systematic or integrative review of literature; 2, experimental (experimental and control groups or cross-over design); 3, quasi-experiemental (non-equivalent control group or repeated measures); 4, pre-experimental (comparison group, or pre-test/post-test design).

^b^Kirkpatrick levels (as adapted by Barr et al., 2000) [5]: 1A, Assessment of students ‘reactions to the teaching programme and methods; 1B, programmatic assessment of satisfaction with strategies and methods; 2A, modification of attitudes and perceptions (learning); 2B, measurement of change in knowledge as an outcome of the teaching event; 3, behaviour changes enacted on-the-job, subsequent to, and attributed to the learning event; 4A, measureable results of the training programme in terms of the system; 4B, benefits to clients or patients; client outcomes.

### Students

Higher academic performance in secondary school was found to predict academic and clinical success; however, older age (postsecondary school) and gender did not contribute to either academic performance or student retention through graduation [6]. Targeted recruitment of qualified students from rural and low-resource settings was a particularly effective strategy for retaining students in vulnerable communities after graduation [7,8]. An applicant’s specific expression of interest in the profession was important to student retention [9]. Student support strategies (for example, financial support and mentoring) offered some advantage to student retention and expanded student diversity [10-12].

Several studies attempted to identify the factors within PSE that promote the preparation of graduates for practice in low-resource, rural and remote settings, and increase the likelihood of their retention in those places. An applicant’s expression of positive attitudes and values toward rural practice as a career choice was directly influential [13]. The promotion of undergraduate clinical learning experiences in rural settings was shown to increase students’ comfort and thus might change their perceptions, attitudes and values about rural and remote practice [14-16].

### Curriculum

Frank et al. explored the definition of the term competency-based education (CBE) [17]. While the authors found no consensus in the literature, the elements of a CBE appeared to be commonly acknowledged, and a competency-based curriculum was identified as one of the building blocks of CBE. Jayasekara, Schultz and McCutcheon explored problem-based learning (PBL) as one strategy for delivery of such a curriculum and identified some positive effects of PBL on knowledge acquisition [18-21]. A few studies attempted to compare the performance of graduates from programmes that used the PBL curriculum approach and reported some favourable findings with respect to impact of a PBL curriculum on improvement of attitudes toward clients, continuity of care, and access to specific services [22-24]. Studies that compared PBL to traditional curricula did not provide incontrovertible evidence to favour the design over any other, but they did not dispute it as a strategy to promote competency-based learning.

No contemporary studies were identified that compared the graduates educated within technical (leading to a certificate of completion) or academic (culminating in at least a baccalaureate degree) programmes of study in terms of competence in the workplace.

Formative and summative assessment of student learning is critical to CBE. A good deal of progress has been made in the development of valid and reliable tools for these assessments [25]. The objective, structured clinical examination (OSCE), which provides an integrated measure of knowledge and skills, was found to be of particular value [26].

### Teachers and preceptors

The preparation and retention of skillful teachers is essential to the effective transfer of knowledge and skills from teachers to students [27-29]. Few studies indicated that a teacher in the health professions must be equally skilled as both a classroom teacher and a clinical preceptor or mentor (an individual who guides learning for one or more students in the clinical setting). Rather, the descriptive literature supports the importance of the preceptor/mentor role as complementary to the role of the academic educator [30]. The role of the preceptor has been particularly well explored, and studies confirm the need for preceptors to develop skills in clinical teaching, identification of student learning needs, assessment of student learning, and prioritizing and time management [31,32].

No studies were found that directly address the issue of an appropriate teacher-to-student or preceptor-to-student ratio. Professional associations or country regulatory authorities sometimes set specific benchmarks and standards for this. A fundamental premise of competency-based education is that students must have both adequate time and sufficient clinical practice opportunities to acquire and demonstrate requisite knowledge and skills. A recent commentary [33] noted that pre-service education for midwives in Africa falls short in terms of adequate numbers of committed and well-informed preceptors to ensure optimal competency-building.

The literature supports the premise that administrative leaders in academic and clinical settings must be strongly supportive of each other’s roles and responsibilities [34,35]. Academic administrators must be cognizant of the impact that clinical training has on the provision of client care. Facility administrators must appreciate the added and long-term value of having students in the clinical setting, as their presence encourages facility providers (both staff and preceptors) to keep up-to-date on the science that is the foundation for their clinical practice.

### Infrastructure and management

Little in the literature speaks to the physical elements of the learning environment (for example, physical space) or teaching/learning tools (for example, textbooks and clinical anatomic models). However, several descriptive studies address elements of the infrastructure that warrant consideration. Modern, Internet-enabled medical libraries, skills and simulation laboratories, and computer laboratories to support computer-aided instruction are promoted as ‘essential enhancements’ to teaching and learning environments for the health occupations and professions [36-40].

Internet-based learning has been extensively studied, and several meta-analyses indicate that it can be as effective as traditional classroom-based learning in terms of student satisfaction and knowledge acquisition [41-45]. It does present challenges for the development and assessment of clinical skills, but these can be offset through the use of simulations and computer-based virtual patients [46,47].

### Clinical practice sites

All students must receive clinical practice learning opportunities in high-quality clinical learning environments, preferably where effective practice can be modeled by highly qualified teachers and preceptors [48,49]. A wide variety of settings may be necessary in order to provide all students the individual learning opportunities that they require, given the lower frequency of occurrence of certain clinical conditions, and/or because of a high volume of students of various health occupations and professions who may be seeking similar experiences in a simultaneous timeframe (particularly in large teaching hospitals). Several systematic reviews offer modest support for the value of two or more health professions learning together in academic and clinical settings, to increase understanding of their complementary skills and contributions [50-52]. A few studies have demonstrated that multidisciplinary learning has positive effects on client outcomes, particularly in terms of better provider-client communication and service utilization [53-55].

### Other influencing factors

Although other environmental factors influence opportunities for students to acquire competence by the time of their graduation and to progress along the continuum from novice to expert practitioner, few higher-tier studies address these factors in the context of pre-service education.

#### Professional regulation

The literature offers only descriptive information about the manner in which government and professional regulations affecting the education and training for the health professions (for example, accreditation standards, clinical practice guidelines, certification of graduates) are designed and implemented, and about their effectiveness in ensuring that schools meet standards and graduates achieve competencies [56,57]. While regulation is widely viewed as essential to public protection, the literature about the relationship between engagement in continued professional development activities and subsequent changes in practice knowledge, skill or behaviour is very useful in any discussion about continued competence and re-licensure, but it offers only an indirect inferential link between pre-service education and professional practice.

#### The context of health service delivery

The degree to which countries succeed in reaching targets for health services coverage is affected not only by the numbers of practitioners, but also by having the right skill mix for national needs and the burden of disease [58,59]. Another critical factor is the degree to which practitioners, including new graduates, are deployed to settings in which this skill mix can be used most effectively in time and place. Studies describing the adverse impacts of underutilization of human resources abound. Again, while the literature is full of studies addressing these and other characteristics of a supportive workplace environment, few studies do so in the context of pre-service education.

### Outcomes and impact

Boelen and Woolard [60] argue that social, economic, cultural and environmental determinants of health must guide the strategic development of educational institutions. The graduates these programmes produce should both possess all of the competencies that are desirable, and also use them in professional practice. It could also be argued that educational programmes have a global accountability, given that they should be responsive to international standards and guidelines, and in light of the international mobility of the health workforce. The expected outcomes and the desired health impact of the high-quality health care services offered by graduates of pre-service education programmes are discussed below.

#### Intermediate outcomes

Although no studies were identified that linked elements of quality PSE to intermediate outcomes such as implementing life-saving practices and demonstrating professional behaviours, many high-quality studies do examine these outcomes in the context of continuing professional education [60-63].

#### Longer-term outcomes

Client satisfaction is an outcome assessed in a number of studies of inter-professional education. These studies generally report positive client responses to receiving team-based care [50]. Similarly, a number of studies assess the impact on satisfaction with care when clients and providers have similar ethnic and cultural backgrounds; clients generally indicate greater satisfaction with care when the provider is perceived as culturally sensitive [64].

The education of a sufficient number of healthcare workers addresses only part of the human resource crisis in many countries. Retention of health practitioners, both within the health occupation or profession for which an individual has been educated and in the location where the individual’s skills are needed, is an equally compelling challenge. Unfortunately, the literature is underdeveloped in terms of how production and retention are linked with PSE.

#### Health impact

Although there are many reports on the positive short- and longer-term outcomes of continuing professional education, very few studies make the link between PSE and these same outcomes, and even fewer examine the impact of PSE on health outcomes such as reduction in adverse events of care, improvement in the quality of life of clients, or reduction of morbidity and mortality. Nevertheless, a logical argument can be made that production of trained and competent staff through high-quality pre-service education and continuing professional development is the foundation required to achieve these desired outcomes.

## Discussion

Fauveau, Sherratt and de Bernis set forth several key areas of work that must be addressed when planning for scale-up of human resources for maternal health [65]. They urge country governments to aim for quality over quantity. They urge a concentrated effort in PSE to address the need for promotion and development of: (1) high levels of technical competence; (2) appropriate curricula that ensure sufficient time for hands-on practical training, leading to competence in basic and emergency clinical tasks; (3) gender sensitivity; (4) excellent interpersonal communication and cultural competencies; and (5) motivation for the job. Based on our experience in PSE for maternal health and on our review of the literature on essential components of PSE, we support the agenda proposed by Fauveau and colleagues. However, good and complete evidence for each component of their agenda is still needed.

The literature review was designed to maximize reflection and discussion. We adopted a very structured grading method for inclusion and exclusion of articles, and involved several reviewers in this process, in order to identify and select objective studies. The literature proved to be rich in discussion of the elements of quality pre-service education but underdeveloped in discussion of proven linkages between PSE and quality healthcare service delivery, which is the intended outcome of competency-based education. The evidence from research already reported in the literature should form the basis of quality PSE programming. At the same time, a research agenda must be undertaken to advance our knowledge of factors essential to quality PSE and to address the gaps in the literature on the relationships and linkages between PSE and health outcomes [66].

## Conclusions

The shortage of health workers in low- and middle-income countries demands an aggressive but structured evidence-based response. Sound pre-service education gives individuals entering the workforce the competencies they need to make an immediate impact as well as a foundation for future professional growth. Our conceptual model reflects the investments that countries must make to develop high-quality pre-service education programmes that are capable of producing competent graduates who are ready to enter the workforce. We urge a parallel and concurrent investment in: (1) developing and strengthening PSE programmes to ensure that they are consistent with current international education standards; (2) ongoing monitoring and evaluation of PSE programme quality and quality improvement initiatives; (3) continuing professional development, to update knowledge and skills of health cadres in an era of rapidly changing science, and to sustain health workers’ commitment to lifelong learning; and (4) long-term basic and operations research designed to demonstrate the value of any country’s investment in pre-service education and its link to practice.

## Abbreviations

CBE: Competency-based education; PBL: Problem-based learning; PSE: Pre-service education; SORT: Strength of recommendation taxonomy.

## Competing interests

The authors declare that they have no competing interests.
